# Conclusions Reached from Personal Experience with Appendicitis

**Published:** 1906-09

**Authors:** Wm. Perrin Nicolson

**Affiliations:** Atlanta, Ga.


ATLANTA

### Journal-Record of Medicine

**Successor to Atlanta Medical and Surgical Journal, Established 1855, and Southern Medical Record, Established 1870.**

OWNED BY THE ATLANTA MEDICAL JOURNAL CO.

**Published Monthly.**

*Official organ Fulton County Medical Society, State Examining Board, Presbyterian Hospital, etc., etc.*

VOL. VIII. SEPTEMBER, 1906. No. 6.

BERNARD WOLFF, M.D., EDITOR, Nos. 319–20 Prudential.

M. B. HUTCHINS, M.D., BUSINESS MANAGER, 1007–1008 Century Bldg.

E. G. BALLENGER, M.D., ASSOCIATE EDITOR AND ASSISTANT MANAGER.

### *ORIGINAL COMMUNICATIONS.*

### CONCLUSIONS REACHED FROM PERSONAL EXPERIENCE WITH APPENDICITIS.*

BY WM. PERRIN NICOLSON, M.D.,

ATLANTA, GA.

Certainly one can not be expected to bring forward anything new in the study of appendicitis, yet it is always useful to us to compare individual methods and exchange individual opinions, as only by such means can we hope to reach any uniform or definite conclusions. As it is the policy of this Society to depart from text-book studies, and to bring into touch our conclusions wrought out of personal labor and individual results, I shall follow this line of thought and endeavor to lay before you some of my positive convictions, reached through a personal experience

*Read before the Fulton County Medical Society.

of more than three hundred operative cases, and certainly a much larger number that did not come to the operating-table.

Perhaps the most important question in reference to appendicitis is that of diagnosis, because this seems, at this late day, to be the stumbling-block in the way with a large number of practicians. In a large majority of cases, diagnosis is easy, provided we recognize the fact that this is the most frequent acute affection of the abdominal cavity. It is a great mistake to attempt to exclude appendicitis and search for other causes when we know that in any acutely painful abdominal seizure, regardless of the location of pain, the presumption is in favor of this trouble. In the language of Deaver, in such cases, with more or less violent pain, accompanied by rise of temperature, with tenderness in the right quadrant of the abdomen, the appendix becomes the defendant, and the burden of proof of innocence rests with it. In my experience, the location of the pain has at first been, exceptionally, over the region of the appendix, and I have seen it more frequently umbilical, left iliac, right or left hypochondriac, epigastric, precordial, lumbar, vaginal, in fact, everywhere else but over the lesion. I do not know of any mistake into which most physicians so often fall as looking for pain in the right ilias region, and frequently you will hear some one say that he would have had no doubt in diagnosis had the pain been in the right side. How many surgeons have seen patients come to the operating-table with the epigastrium blistered with mustard or other counter-irritants, because the pain was located at this point and associated with vomiting. It should be remembered always that while the pain is at first most variable in locality, tenderness is always constant over the region of the appendix, though in the very early stage, it may be limited to a very circumscribed area and only be elicited by deep pressure, and it may be, as seen in three cases by me, absent even on deep pressure at the first visit, but at the next, apparent and the diagnosis undoubted. I am sure that spasmodic colic is a rare affection in grown persons, and such a diagnosis is always subject to grave question.

In my experience, the diseases with which appendicitis has been most easily confounded have been acute cholecystitis, typhoid perforation, acute pyelitis of the right kidney, and inflamed Meckel's diverticulum. As these conditions before mentioned require surgical intervention, the distinction is not absolutely necessary. Experience leads me to believe that he who, when in doubt, gives the benefit of such doubt in favor of appendicitis, will find far less cause for regrets in future than he who does the opposite. The importance of accurate diagnosis in the earliest possible stage can not be questioned, and at this time, it is made with more difficulty than later. When the classical tumor has formed, and the symptoms are proclaimed that he who runs may read, often the golden opportunity has passed. For early diagnosis, then, given a pain at any point in the abdomen, accompanied by nausea or vomiting, with tenderness in the right lower quadrant, accompanied by rising temperature and with beginning muscular rigidity, one is justified in making a diagnosis of appendicitis, and he will rarely have occasion to recede from it.

*Treatment.*—Appendicitis is essentially a surgical disease, and for its cure, is amenable only to surgery. True, many attacks are passed through in safety by medical treatment, and this is a fact well known by both the profession and the laity. Undoubtedly, many attacks are fatal from the beginning, except when relieved by surgery, and these are absolutely unaffected by any known medical treatment. I would lay down the proposition that the treatment of appendicitis depends entirely upon the stage at which it was seen and the history of the case. He who applies the same routine treatment at all stages, even though that be surgical, will not attain to the best results, and will certainly have a high mortality rate of which to boast. Seen within the first twenty-four to forty-eight hours, the ideal treatment is surgical, which consists of the removal at the earliest possible moment, be it night or day, because every hour adds to the chances of mortality. This period constitutes what is known as the period of safety, and every day added to it adds to the surgical mortality. Should the patient decline operation, or circumstances are such that operation can not be done, what means are most effectual in controlling the inflammation? With me, nothing can be compared to the application of the ice-bag, which is called in Philadelphia "treatment by cold storage." Hot applications have been entirely unsatisfactory with me. Mild purgation in the early stage, consisting of calomel followed by salines, are effectual in reducing the inflammation, and undoubtedly, as stated above, many cases will pass on to a speedy convalescence under this treatment, and to a feeling of peaceful security, from which the patient will some day be rudely awakened by another attack. While these remarks apply to those attacks of minor severity, where no great amount is lost by watching and waiting, they do not apply to those explosive and violent cases which, from the beginning, bear all the marks of intensity. For such, there is no remedy but immediate operation, as soon as the diagnosis is made, and the patient can be gotten ready.

Where an attempt is made to control appendicitis by the ice-bag, unless its application is followed in a few hours by an amelioration of all the cardinal symptoms, we may be assured that the case will pass on to suppuration or gangrene, and the operation should be done within the limit of safety. No case is doing well unless there is a continual subsidence of the pain, tenderness, temperature and pulse, and the failure of either one of these to yield is a proof that things are not going well. It is at this point that the use of opiates is so disastrous in results, because the pain is the great guide in most instances, and its cessation, without the use of anodynes, is nearly always a favorable sign. In fact, it has been my observation that the use of opiates from the early stages of appendicitis is to be deprecated most emphatically, unless it is confined to one initial dose when the patient is first seen, and is suffering very intensely, in that it puts at rest the nervous system for a while and gives us an opportunity to look into matters more thoroughly.

My experience, then, has been that even where an attempt is made to control the initial stages of the disease, either on account of the attitude of the patient as regards operation, or where there are not suitable environments for the proper performance of the same, we can at least keep a watchful eye while using medical means, and at the same time interfere in time to save life.

The cases that tax the soul of the surgeon is the determination of what course to pursue with those seen after the first forty-eight or seventy-two hours have passed. I know many claim that we should operate when we see them, regardless of circumstances, and their position reminds me of the story that the late Dr. Briggs told of himself, in a paper upon ovarian tumors, which was translated into German; they made him say that he operated when they let him in. The work of all surgeons will show that operations performed between the third and tenth day are very dangerous, and I am confident that the average man or woman who has resisted the disease for four days, will pass through the attack, and the abscess will take care of itself. This is eminently true where there is a well-marked tumor over the appendix. Any one who will attempt to unravel one of the tumors will get some idea of the nature of the wall which has been built up. Personally, I have never seen a well-walled off abscess opening into the peritoneal cavity. In the immense majority of cases they empty themselves into the intestines, though occasionally they point externally. The advocates of immediate evacuation of pus whenever found stand upon apparently plausible ground, but I believe it to be wrong as applying to these conditions. The teaching of Deaver and Price and others that we shall go in, evacuate the abscess, break up all adhesion, and remove the appendix and drain, may be successful in their hands, but for the average surgeon I believe it to be a most hazardous procedure.

In average hands, I am sure that drainage, without extensive attempts to remove the appendix, is the safe operation, and the appendix can be removed later if necessary, and most patients are more willing to submit to two harmless operations than to one very dangerous one. Simple drainage usually means leaving in place a bad appendix, complicated with a hernia, and if an abscess would empty itself, under the watchful eye of its surgeon, the patient will be spared one operation. In appendix abscesses then, I have advocated an expectant line of treatment, keeping ever in mind the relation of the cardinal points already mentioned, and being ready at any time to interfere when evidence shows that nature is losing in the fight. Should the patient develop a condition of sepsis, there should be no hesitation in draining the abscess at once, but the operation performed later in the disease is far safer than one during the first formation of the abscess.

*Chronic Appendicitis.*—It is my firm conviction that more reflex disturbances and chronic invalidism from abdominal irritation and faulty assimilation, result from chronic appendicitis than any other single cause. Chronic indigestion, especially of an intestinal type, finds here certainly one of its most potent causative factors. This is frequently true in cases where the patient has never given a history of any well-marked attacks, and the condition seems one of interstitial inflammation with extreme hyperesthesia. In all cases of chronic invalidism, no investigation is complete without a thorough examination of the appendix. Sometimes, the tenderness is confined immediately to the organ itself, and can be only elicited by point pressure with one finger, while again there may be general abdominal tenderness. Chronic appendicitis is very common in women, and is not unusually confounded with right ovarian disease, but in my experience the tenderness from ovarian disease has never extended as high as McBurney's point. Numbers of women have suffered a loss of the ovaries from which no relief was obtained, and the abdominal pain and reflexes remained, only to disappear when the appendix was removed later. There is a case in this city where a young lady lost both ovaries at the age of seventeen, passed through years of torture, and was finally relieved only when I removed her appendix five years later. I have many times seen intense dysmenorrhea cured by removing a damaged appendix, and, in fact, I believe that many patients suffer from this condition where it is due solely to chronic appendicitis. Had women no ovaries, we would find no exact diagnosis in such cases. I am convinced that reflexes through the solar plexus do not belong to ovarian disease, and the cause in such cases should be sought for in the appendix and other organs. I have recently seen a patient who said she had been operated upon by a gynecologist, who told her a general surgeon would have pronounced her trouble appendicitis; he did some pelvic operation for the relief of her pain, nervousness and tenderness, but her condition remained unimproved, because he backed up his judgment by refusing to remove her appendix, even though it was within his immediate reach. While I do not at all dispute the fact that there was apparent pelvic disease, I believe there was probably also a diseased appendix, which did not give very marked appearance of inflammatory trouble, and yet which is now the focus from which the trouble goes out.

*Operation.*—I shall have but little to say about the technic of operation, because there is little to bring out with which the profession is not familiar, and because of the fact that different methods of operating give good results in different hands.

As regards the incision, I have learned not to make it through the rectus muscle, but well out to the spinous process of the ileum. I do this because it is the line of natural drainage in acute cases, and in chronic cases, the appendix is most frequently found here, and can be removed through a smaller incision. The incision here in acute cases saves the necessity of a scab wound, recommended by many operators, for the purpose of draining in acute cases. In chronic cases, I know of no reason for departing from a McBurney incision, for experience will enable one to remove almost any appendix through the room afforded by this incision. The tendency with me has been toward small incisions, as they do not damage the abdominal wall. Operations within the first forty-eight hours require a smaller incision than those later.

The longer I operate, I believe, the more I drain. The dictum sent forth from the Hopkins Hospital that drainage is a confession of a weak technique, has caused the death of hundreds of patients in the hands of surgeons who should have drained. While I have faith in the peritoneum, I have much more when I have given it an opportunity to extrude the infectious material by means of a drain. I drain practically all acute cases, and all cases of operation done soon after an attack, and in those where I tear up many adhesions above the appendix. The small drain can be brought out at one angle of the wound, closed in layers, or through the crossing point of a gridiron incision; it is often astonishing how much serum will be delivered by a small wick. I have never removed a gauze drain under four days, and I do not believe that any one will do so who has ever had a drain pulled out of himself.

In handling the stump of the appendix, I have within the last fifty or seventy-five operations returned to the old method of simply tying off the appendix, cutting it away, and dropping off the stump after tying it with a cut mescoappendix. In closing wounds after acute cases within the first forty-eight hours, I am in the habit of closing the wound in layers with catgut, except one angle, and of supporting the wound with silkwormgut sutures.

### DISCUSSION OF DR. NICOLSON'S PAPER.

*Dr. F. W. McRae:* In the main I agree with Dr. Nicolson's views as set forth in his excellent paper, but there are a few points with which I must take issue.

I do not think we can place much reliance on a rising temperature. I consider the temperature of very little value in making a prognosis. I am also very much afraid of the cases where there is complete cessation of pain which has been severe for several hours, as such cessation, in my experience, has usually indicated either perforation or death of the appendix—as simply the calm before the storm. We must differentiate between acute thin-walled abscesses and those of a more chronic character. I am afraid of abscess cases.

Where there is a probability of having to drain, in very acute cases, I do a transverse operation, as described in my paper read at Augusta meeting. I always drain in acute cases when in doubt. I do not think I have ever regretted draining, but I have regretted not draining.

*Dr. H. F. Harris:* As regards Dr. Nicolson's statement that chronic appendicitis is very common in women, I desire to say that membranous colitis, which is very frequent in women, is often mistaken for appendicitis. I have known of three instances where patients with this disease were operated on and their appendix removed with absolutely no good effect.

*Dr. Kime:* You all know where I stand on this question. All surgeons must admit if nature is given a good chance she will carry many cases through severe attacks even with perforation, pus formation, etc.

Cases are on record where the appendix was even destroyed. Not long since I had such a case myself. I removed piece of omentum the size of hand to get to site of trouble, separated adhesion to small intestine and closed in same, on reaching point where appendix should have been, found a tablespoonful or more of degenerated, broken-down tissue; cleaned out same, applied oxygen solution pure followed with pure carbolic acid, then alcohol, and closed up surface one and one-half by two inches with catgut, and then closed abdomen without drainage. Patient did as well as any I ever operated upon, and made complete recovery.

The case had first attack six years previous. Husband died two years ago of tuberculosis—tubercular germs were found in debris removed.

I indorse Dr. Nicolson's paper and am very glad to commend the conservative stand he has taken as to operative work in appendicitis.

